# FKBP12 is a predictive biomarker for efficacy of anthracycline-based chemotherapy in breast cancer

**DOI:** 10.1007/s00280-019-03923-1

**Published:** 2019-08-19

**Authors:** Mingyou Xing, Jun Wang, Qin Yang, Yu Wang, Jiansha Li, Jing Xiong, Sheng Zhou

**Affiliations:** 1 Institute of Pathology, Tongji Hospital, Tongji Medical College, Huazhong University of Science and Technology, Wuhan, 430030 China; 2 Department of Infectious Disease, Tongji Hospital, Tongji Medical College, Huazhong University of Science and Technology, Wuhan, 430030 China

**Keywords:** Breast cancer, FKBP12, MDM2, Prognosis, Chemoresistance

## Abstract

**Background:**

FK506-binding protein 12 (FKBP12) is abundant, ubiquitously expressed cytoplasmic protein with multiple functions in cell signaling transduction. Recently, we reported a novel function for FKBP12 in oncoprotein mouse double minute 2 (MDM2) self-ubiquitination and degradation, which greatly enhanced the sensitivity of cancer cells to chemotherapy. However, the clinical relevance remains unclear.

**Methods:**

An immunohistochemical analysis of FKBP12 expression was performed in a cohort of 524 patients with invasive breast cancer. The correlations of FKBP12 expression with patient survival and chemoresponse were statistically analyzed. MDA-MB-468 cells were transfected with FKBP12 siRNA or Myc-tagged FKBP12, and then, the tumor cells were treated with doxorubicin followed by western blot, cell viability, and apoptosis assay.

**Results:**

The expression of FKBP12 was decreased in breast cancer tissues, and there was a significant correlation between FKBP12 loss and MDM2 overexpression. Furthermore, FKBP12 loss was specifically correlated with poor prognosis and increased resistance to anthracycline-based chemotherapy. Kaplan–Meier survival analysis showed that overall survival (OS) and disease-free survival (DFS) were both significantly lower in the low FKBP12 expression group than those in the high FKBP12 expression group. In patients treated with anthracycline-based preoperative chemotherapy, low FKBP12 expression patients had a significant lower rate of pathologic complete response (pCR). Importantly, these results seemed to be driven mainly by MDM2. These observations were especially prominent in the MDM2-positive subgroup. Univariate and multivariate analyses revealed that FKBP12 loss was an independent factor for predicting prognosis and pCR. In in vitro assay, FKBP12 silence led to significant upregulation of MDM2. Accordingly, MDA-MB-468 cells with FKBP12 silence were less responsive to doxorubicin-induced cytotoxic and apoptotic effect. In contrast, in FKBP12-transfected MDA-MB-468 cells, MDM2 was more greatly inhibited by doxorubicin, resulting in greater cytotoxic and apoptotic effect.

**Conclusions:**

We propose that FKBP12 loss, which can be enhanced by MDM2 overexpression, predicts poor prognosis and chemoresistance. Increasing the expression of FKBP12 may be a valuable strategy to add to anthracycline-based chemotherapy, especially in MDM2-overexpressed patients.

## Background

Chemotherapy remains the mainstay for human breast cancer treatment. Especially for triple-negative breast cancer (TNBC), with no drug-targetable receptors, cytotoxic chemotherapy is the only recommended therapeutic regimen [1–3]. However, only certain patients respond well to this cytotoxic chemotherapy. Therefore, the treatment of breast cancer, especially TNBC, is a significant challenge. Reliable biomarkers that predict tumor response to chemotherapy are quite valuable when making treatment decisions, and new therapeutic strategies against the corresponding molecular pathways associated with chemoresistance are urgently required to improve clinical outcome.

FK506-binding protein 12 (FKBP12) is a 12-kDa protein that is abundantly and ubiquitously expressed mainly in cytosol and possesses peptidyl prolyl *cis-trans* isomerase activity [4]. It can bind the immunosuppressants FK506 and rapamycin. When bound to FK506, FKBP12 forms a ternary complex with calcineurin to inhibit the serine/threonine phosphatase activity of calcineurin and interfere with the signal transduction in activated T lymphocytes [5, 6]. In complex with rapamycin, FKBP12 interacts with mammalian target of rapamycin (mTOR) and inhibits its roles in regulating cell growth and cancer progression [7, 8]. In the absence of FK506, FKBP12 binds to other different cellular receptors or targets such as ryanodine receptor (RyR), which is one of the major calcium release channels in the sarcoplasmic and endoplasmic reticula. Interaction between FKBP12 and RyR stabilizes RyR channel and modulates channel gating by increasing channel full conductance levels and mean open time [9]. FKBP12 has also been shown to interact with transforming growth factor-β type I receptor (TGF-βRI) to inhibit receptor-mediated signal transduction [10]. In addition, FKBP12 has an inhibitory effect on the cellular activity of epidermal growth factor receptor (EGFR) by modulating the receptor’s phosphorylation status [11].

Recently, we reported a novel function for FKBP12 in oncoprotein mouse double minute 2 (MDM2) self-ubiquitination and degradation, which greatly enhanced the sensitivity of cancer cells to chemotherapy [12]. Specifically, FKBP12 binds to the C-terminal RING domain of MDM2 protein to disrupt heterodimer formation with MDM4 and induces its E3 ligase activity for self-ubiquitination. When cancer cells are subjected to doxorubicin treatment, the increased expression of MDM2 following activation of p53 is also markedly inhibited by FKBP12. This is because p53 induces MDM2 translocation from the nucleus to the cytoplasm, where facilitates FKBP12/MDM2 interaction. FKBP12-mediated degradation of MDM2 confers continuing and constitutive activation of p53, suppression of XIAP, and consequent sensitization of cancer cells to the cytotoxic and apoptotic effects of doxorubicin. However, the clinical relevance of FKBP12 remains unclear. As chemotherapy is still the mainstay of treatment for human breast cancer, we herein examined the expression of FKBP12 by immunohistochemistry in breast cancer. We revealed that there was indeed a significant correlation between FKBP12 and MDM2 expression and that the expression level of FKBP12 in cancer tissue might predict prognosis and response to chemotherapy.

## Materials and methods

### Patients

This study was approved by the Ethics Committee of Tongji Hospital, Huazhong University of Science and Technology. A cohort of 524 patients with invasive breast cancer was included in this study. All patients were diagnosed and treated at Tongji Hospital between September 2007 and January 2012. The patients ranged from 32 to 78 years of age (median, 56 years). Detailed clinicopathologic characteristics of these patients are summarized in Table 1. Tumor was graded according to a modified Bloom–Richardson scoring system. Clinicopathologic staging was based on the American Joint Committee on Cancer (AJCC) Cancer Staging Manual. The Nottingham prognostic index (*NPI*), which is calculated using the size of the lesion (*S*), the number of involved lymph nodes (*N*), and the grade of the tumor (*G*), was used to determine patient prognosis (*NPI* = (0.2 × *S*) + *N* + *G*). According to gene expression, patients were divided into the following three distinct molecular subtypes: Luminal (ER+ and HER2−); HER2+; and TNBC (ER−, PR−, and HER2−). Follow-up data were collected from clinical records, ranging from 5 to 72 months post-diagnosis (median, 48 months). Overall survival (OS) was the period from diagnosis to the date of breast cancer-related death, while disease-free survival (DFS) was the period from diagnosis to the date of breast cancer-derived relapse or metastasis.

**Table 1:** Clinicopathologic variables in relation to FKBP12 and MDM2 immunoreactivity in invasive breast cancer and TNBC

Variable	Total (*n *= 524)	FKBP12 high, no. (%)	FKBP12 low, no. (%)	*P* value	MDM2 high, no. (%)	MDM2 low, sno. (%)	*P* value
Age							
< 56	313	85 (27.2)	228 (72.8)	0.748	189 (60.4)	124 (39.6)	0.471
≥ 56	211	60 (28.4)	151 (71.6)		134 (63.5)	77 (36.5)	
Menopausal status							
Premenopausal	265	81 (30.6)	184 (69.4)	0.134	156 (58.9)	109 (41.1)	0.187
Postmenopausal	259	64 (24.7)	195 (75.3)		167 (64.5)	92 (35.5)	
Size-TNM							
pT1	188	59 (31.4)	129 (68.6)	0.109	106 (56.4)	82 (43.6)	0.112
pT2 + pTx	252	70 (27.8)	182 (72.2)		159 (63.1)	93 (36.9)	
pT3 + pT4	84	16 (19.0)	68 (81.0)		58 (69.0)	26 (31.0)	
Histologic grade							
Grade I	97	33 (34.0)	64 (66.0)	0.264	48 (49.5)	49 (50.5)	0.015
Grade II	217	61 (28.1)	156 (71.9)		135 (62.2)	82 (37.8)	
Grade III	210	51 (24.3)	159 (75.7)		140 (66.7)	70 (33.3)	
LN metastasis							
Positive	319	83 (26.0)	236 (74.0)	0.291	204 (63.9)	115 (36.1)	0.175
Negative	205	62 (30.2)	143 (69.8)		119 (58.0)	86 (42.0)	
*NPI*							
< 3.4	180	57 (31.7)	123 (68.3)	0.107	84 (46.7)	96 (53.3)	< 0.001
3.4–5.4	269	74 (27.5)	195 (72.5)		189 (70.3)	80 (29.7)	
> 5.4	75	14 (18.7)	61 (81.3)		50 (66.7)	25 (33.3)	
MIB-1 (%)							
< 10	204	63 (30.9)	141 (69.1)	0.134	117 (57.4)	87 (42.6)	0.028
10–30	254	70 (27.6)	184 (72.4)		171 (67.3)	83 (32.7)	
> 30	66	12 (18.2)	54 (81.8)		35 (53.0)	31 (47.0)	
ERα							
Positive	343	107 (31.2)	236 (68.8)	0.013	200 (58.3)	143 (41.7)	0.031
Negative	181	38 (21.0)	143 (79.0)		123 (68.0)	58 (32.0)	
PR							
Positive	319	98 (30.7)	221 (69.3)	0.052	187 (58.6)	132 (41.4)	0.076
Negative	205	47 (22.9)	158 (77.1)		136 (66.3)	69 (33.7)	
HER2 amplification							
Positive	103	21 (20.4)	82 (79.6)	0.065	71 (68.9)	32 (31.1)	0.090
Negative	421	124 (29.5)	297 (70.5)		252 (59.9)	169 (40.1)	
Hormone therapy							
No	182	41 (22.5)	141 (77.5)	0.120	121 (66.5)	61 (33.5)	0.114
Yes (Tamoxifen)	332	102 (30.7)	230 (69.3)		198 (59.6)	134 (40.4)	
Unknown	10	2 (20.0)	8 (80.0)		4 (40.0)	6 (60.0)	
Chemotherapy							
No	31	9 (29.0)	22 (71.0)	0.235	19 (61.3)	12 (38.7)	0.118
Yes (FEC)	469	133 (28.4)	336 (71.6)		294 (62.7)	175 (37.3)	
Unknown	24	3 (12.5)	21 (87.5)		10 (41.7)	14 (58.3)	
Recurrence							
No	387	115 (29.7)	272 (70.3)	0.079	228 (58.9)	159 (41.1)	0.031
Yes	137	30 (21.9)	107 (78.1)		95 (69.3)	42 (30.7)	
MDM2 expression							
High	323	81 (25.1)	242 (74.9)	0.092			
Low	201	64 (31.8)	137 (68.2)				
FKBP12 expression							
High	145				81 (55.9)	64 (44.1)	0.092
Low	379				242 (63.9)	137 (36.1)	
Molecular subtype							
Luminal (non HER2+)	334	102 (30.5)	232 (69.5)	0.144	196 (58.7)	138 (41.3)	0.171
HER2+	102	24 (23.5)	78 (76.5)		67 (65.7)	35 (34.3)	
TNBC	88	19 (21.6)	69 (78.4)		60 (68.2)	28 (31.8)	

Variable	TNBC (*n *= 88)	FKBP12 high, no. (%)	FKBP12 low, no. (%)	*P* value	MDM2 high, no. (%)	MDM2 low, sno. (%)	*P* value
MDM2 high	60	7 (11.7)	53 (88.3)	0.001			
MDM2 low	28	12 (42.9)	16 (57.1)				
FKBP12 high	19				7 (36.8)	12 (63.2)	0.001
FKBP12 low	69				53 (76.8)	16 (23.2)	

*LN* lymph node, *NPI* Nottingham Prognostic Index, *ERα* estrogen receptor, *PR* progesterone receptor, *HER2* human epidermal growth factor receptor 2, *FEC* 5-fluorouracil + epirubicin + cyclophosphamide, *TNBC* triple-negative breast cancer

Of the 524 invasive breast cancer patients, 166 (including 67 TNBC) patients received six cycles of anthracycline-based neoadjuvant chemotherapy (FEC 100 regimen: 5-florouracil 500 mg/m^2^, epirubicin 100 mg/m^2^, cyclophosphamide 500 mg/m^2^, once every 21 days). A core needle biopsy was performed prior to chemotherapy for pathologic diagnosis and other biologic evaluation. Patients underwent mastectomy and axillary lymph-node excision or breast-conserving surgery to assess tumor response about 4 weeks after the sixth cycle of chemotherapy. Pathologic complete remission (pCR) was defined as disappearance of invasive tumor lesion in the surgically removed breast and axillary lymph nodes after chemotherapy.

### Immunohistochemistry

The formalin-fixed paraffin-embedded archival tissue block was cut into 4-μm-thick tissue sections. Only tissue samples collected prior to chemotherapy were eligible. The sections were deparaffinized in xylene and subsequently rehydrated through a graded series of ethanol. Endogenous peroxidase was blocked in 3% hydrogen peroxide for 15 min. Antigen retrieval was performed in 0.01 M citrate buffer (pH 6.0) at 100 °C for 20 min. Immunohistochemistry was performed by Envision method. The sections were first incubated with the primary antibody anti-FKBP12 (N-19; 1:100; Santa Cruz Biotechnology, Santa Cruz, CA, USA) or anti-MDM2 (SMP14; 1:200; Sigma-Aldrich, St. Louis, MO, USA) at 4 °C overnight. Negative and blank control samples were run accordingly. After being rinsed three times in PBS, secondary antibody (Envision, Anti-Goat/Mouse-HRP, DAKO, Glostrup, Denmark) was applied to all sections and incubated at 37 °C for 1 h, followed by three PBS rinses. Finally, the sections were developed with diaminobenzidine (DAB) and counterstained with hematoxylin.

Staining results were evaluated by three independent pathologists blinded to the patient details. FKBP12 shows cytoplasmic staining, while MDM2 shows nuclear staining with or without cytoplasmic staining. FKBP12 and MDM2 expression were scored using a modified histochemical score (H-score). H-score includes both the intensity of staining and the percentage of stained cells. The intensity of staining was scored 0–3 (0 = no staining, 1 = weak staining, 2 = moderate staining, and 3 = strong staining). The percentage of stained cells was estimated (0–100%). H-score (range from 0 to 300) was calculated by multiplication of the two indices. Using a cut-off score of 100, FKBP12 or MDM2 expression was categorized into two groups: low expression (H-score < 100) and high expression (H-score ≥ 100).

### Cell culture, plasmid transfection, and doxorubicin treatment

MDA-MB-468 cells were obtained from the China Center for Type Culture Collection (CCTCC). siFKBP12 and Myc-tagged FKBP12 plasmids were kindly provided by Prof. Muxiang Zhou (Emory University School of Medicine, Atlanta, GA, USA). Doxorubicin was purchased from Sigma-Aldrich. MDA-MB-468 cells were transfected with FKBP12 siRNA or Myc-tagged FKBP12 using Lipofectamine 2000 transfection kit from Invitrogen (CarIsbad, CA, USA), followed by treatment with different concentrations of doxorubicin. The cells were collected for western blot, cell viability, and apoptosis assay.

### Western blot

Western blot, performed by standard procedures, was used to measure the protein expression levels of FKBP12 and MDM2 in tumor cells. The following antibodies were used: anti-FKBP12 (N-19; 1:1000; Santa Cruz Biotechnology), anti-Myc (9B11; 1:1000; Cell Signaling Technology, Danvers, MA, USA), and anti-MDM2 (SMP14; 1:1000; Sigma-Aldrich). GAPDH served as an internal control.

### WST-1 assay

Cell viability was detected by WST-1 assay. Cells with FKBP12 siRNA or Myc-tagged FKBP12 transfection were grown in 96-well plates, treated with different concentrations of doxorubicin for 24 h, and incubated with WST-1 reagent (Roche, Mannheim, Germany) for an additional 4 h. The optical density was read using a microplate reader.

### Flow cytometry

Cell apoptosis was detected by flow cytometry. Cells with FKBP12 siRNA or Myc-tagged FKBP12 transfection and doxorubicin treatment were stained with FITC-Annexin V and propidium iodide (BD Pharmingen, San Diego, CA, USA). Stained cells were detected on a FACScan flow cytometer.

### Statistical analysis

All data were analyzed using SPSS statistical software. The Chi-square and Fisher’s exact tests were used to examine the correlations between FKBP12 or MDM2 expression and different clinicopathologic variables. The Spearman rank correlation coefficient test was used to evaluate the correlations between FKBP12 and MDM2 in breast cancer tissue. Survival curves were plotted using the Kaplan–Meier method, and the significance of observed differences was determined using the log-rank test. The influence of each variable on survival was tested with the Cox proportional hazard regression model. *P* < 0.05 was considered statistically significant.

## Results

### FKBP12 and MDM2 protein expression pattern in breast cancer tissue

**Fig. 1:**
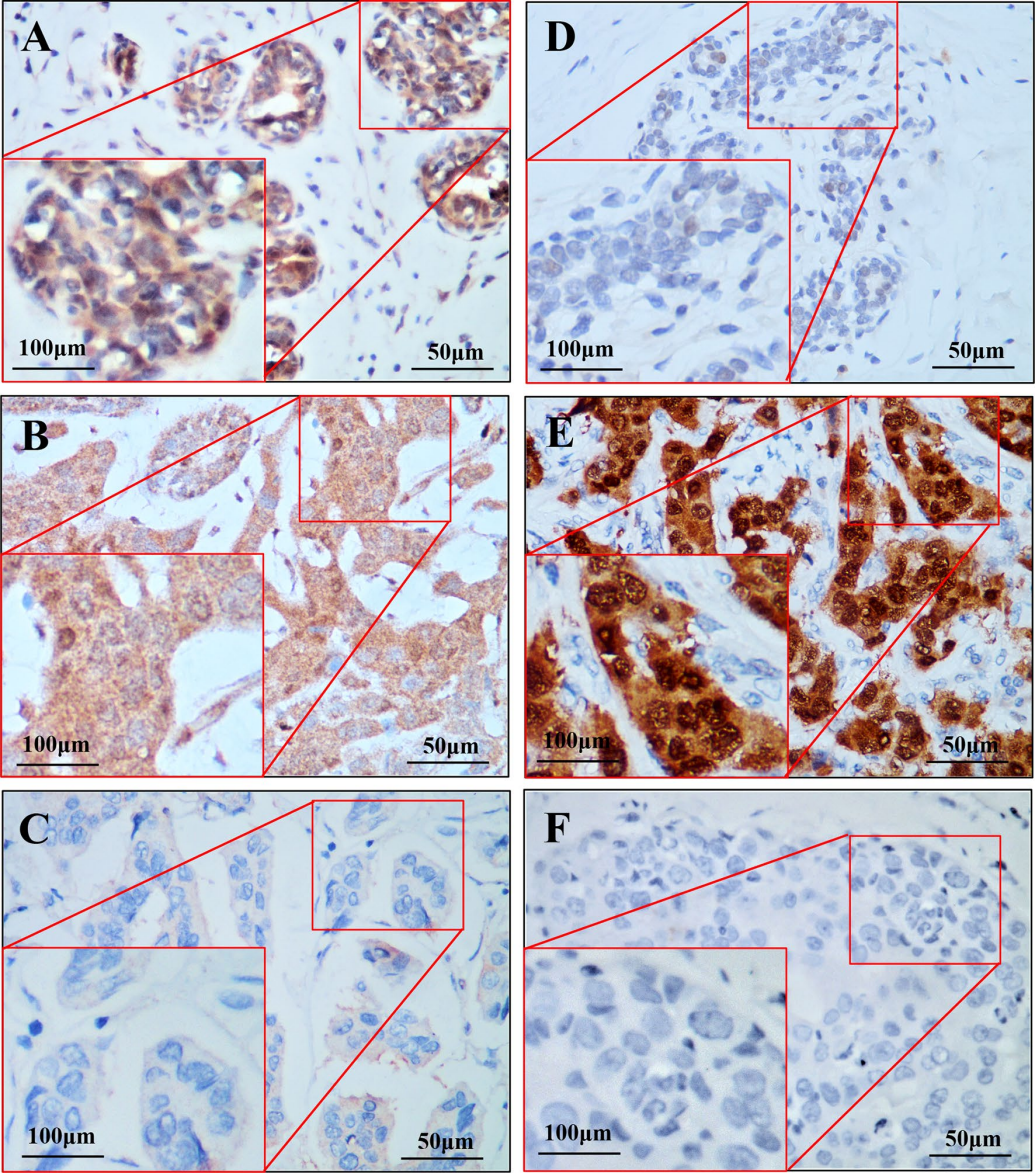
Representative images of immunohistochemical staining for FKBP12 and MDM2 protein. **a** FKBP12-positive staining in benign breast tissue. **b** FKBP12 high staining in invasive breast cancer. **c** FKBP12 extremely low staining in invasive breast cancer. **d** MDM2 low staining in benign breast tissue. **e** MDM2 high staining in invasive breast cancer. **f** MDM2-negative staining in invasive breast cancer

FKBP12 protein expression was observed in the cytoplasm of epithelial cells of normal breast ducts and lobules. However, FKBP12 protein expression was decreased in breast cancer tissues. Of the 524 invasive breast cancers, 145 (27.7%) showed high immunoreactivity (moderate-to-strong staining), while 238 (45.4%) showed low immunoreactivity (weak staining) and 141 (26.9%) showed an absence of staining. MDM2 protein showed absent or weak expression in normal breast tissues. However, immunopositive reaction for MDM2 was detected in 323 (61.6%) of the 524 invasive breast cancers. Subcellular localization of MDM2 protein in cancer cells was predominantly nuclear with or without cytoplasmic. Representative images of FKBP12 and MDM2 expression are presented in Fig. 1.

### FKBP12 and MDM2 expression correlated with clinicopathologic parameters

We correlated FKBP12 and MDM2 expression to patient clinicopathologic characteristics such as age at diagnosis, tumor size, histologic grade, lymph-node metastatic status, *NPI*, proliferation rate of breast cancer cells (Index of MIB-1), ER, PR, HER2, and molecular subtype. The correlations are summarized in Table 1. Loss of FKBP12 was significantly correlated with ER negativity (*P* = 0.013). MDM2 overexpression was also significantly correlated with ER negativity (*P* = 0.031). There was no significant correlation found between FKBP12 or MDM2 expression and HER2 amplification (*P* > 0.05). According to gene expression, patients were divided into three different molecular subtypes: luminal (ER+ and HER2−); HER2+; and TNBC (ER−, PR−, and HER2−). Our results showed that FKBP12 or MDM2 expression was not differently represented in any of these particular molecular subtypes (*P* > 0.05). In addition, MDM2 overexpression was found to be significantly correlated with higher histologic grade (*P* = 0.015), higher *NPI* (*P* < 0.001), higher index of MIB-1 (*P* = 0.028), and recurrence (*P* = 0.031). No significant correlation occurred between FKBP12 expression and these parameters (*P* > 0.05).

### FKBP12 loss correlated with MDM2 overexpression

Using Spearman rank correlation coefficient test, we studied the correlation of FKBP12 with MDM2. There was a trend toward correlation of FKBP12 loss with MDM2 overexpression, but the difference did not reach statistical significance in the overall population (*n *= 524, *P *>0.05) (Table 1). However, in TNBC patients, we found a significant correlation between FKBP12 loss and MDM2 overexpression (*n *= 88, *P *= 0.001) (Table 1).

### Correlation of FKBP12 expression with clinical outcome

**Fig. 2:**
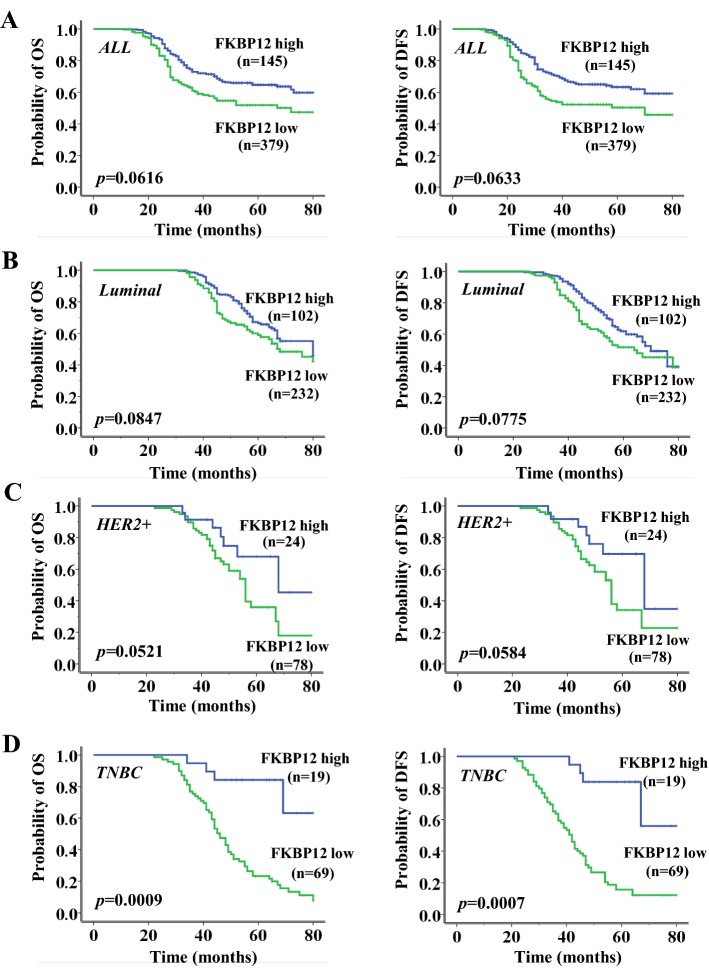
Overall survival and disease-free survival curves according to FKBP12 expression in all patients (**a**), luminal patients (**b**), HER2+ patients (**c**), and TNBC patients (**d**)

**Fig. 3:**
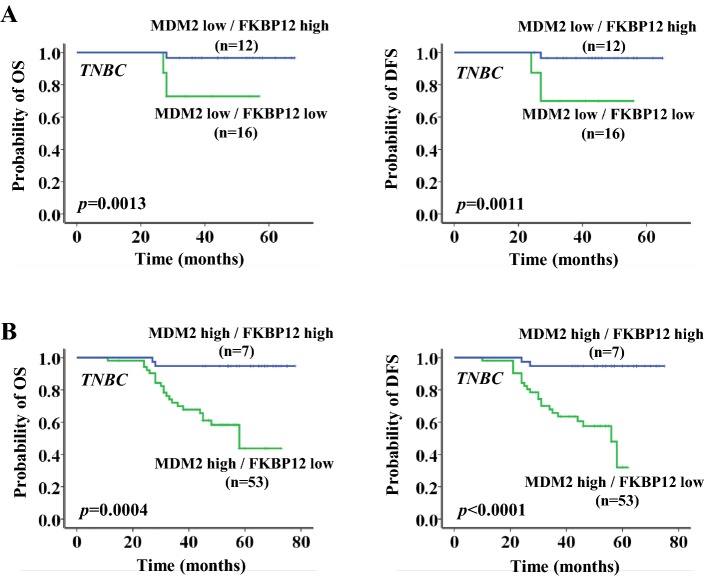
Overall survival and disease-free survival curves according to FKBP12 expression in MDM2-negative TNBC patients (**a**) and MDM2-positive TNBC patients (**b**)

Next, we investigated the correlation of FKBP12 expression with clinical outcome to study the clinical significance of FKBP12. Kaplan–Meier survival analysis was performed in the overall population and different molecular subtypes of breast cancer. As shown in Fig. 2, FKBP12 loss indicated a worse clinical outcome. OS and DFS were both significantly lower in the low FKBP12 expression group than those in the high FKBP12 expression group. FKBP12 loss was significantly correlated with worse OS and DFS in TNBC patients (*n *= 88, *P *= 0.0009 and *P *= 0.0007, respectively) (Fig. 2d), but not in the overall population (*n *= 524, *P *>0.05) (Fig. 2a), in patients with luminal breast cancer (*n *= 334, *P *>0.05) (Fig. 2b), or in patients with HER2+ breast cancer (*n *= 102, *P *>0.05) (Fig. 2c). Interestingly, the result seemed to be driven mainly by MDM2. This observation was especially prominent in the MDM2-positive subgroup (OS, *P *= 0.0004 for MDM2-positive subgroup and *P *= 0.0013 for MDM2-negative subgroup; DFS, *P *< 0.0001 for MDM2-positive subgroup and *P *= 0.0011 for MDM2-negative subgroup) (Fig. 3a, b). Cox multivariate analysis revealed that, independent of lymph-node involvement and MDM2 overexpression, FKBP12 loss was an independent prognostic factor for TNBC patients in OS and DFS (*P *<0.001 and *P *<0.001, respectively) (Table 2).

**Table 2:** Multivariate Cox model analysis of disease-free survival and overall survival in TNBC

Variable	Disease-free survival			Overall survival		
	Hazard ratio	95% CI	*P* value	Hazard ratio	95% CI	*P* value
Age (< 56 vs ≥ 56)	0.834	0.433–1.748	0.776	0.903	0.389–2.124	0.786
Menopausal status (pre. vs post.)	0.687	0.325–1.975	0.477	0.525	0.223–1.867	0.335
Size (pT1-2 vs pT3-4)	0.659	0.236–1.682	0.065	0.387	0.233–1.407	0.059
Histological grade (I–II vs III)	0.412	0.237–1.531	0.158	0.704	0.498–1.994	0.086
LN metastasis (Posi. vs Neg.)	0.627	0.247–1.033	0.014	0.754	0.162–1.228	0.019
*NPI* (< 3.4 vs ≥ 3.4)	1.632	1.114–3.152	0.113	0.603	0.327–1.178	0.076
MIB (< 10% vs ≥ 10%)	0.631	0.443–0.987	0.069	0.836	0.530–1.625	0.097
FKBP12 (high vs low)	0.512	0.201–0.739	< 0.001	0.317	0.186–0.586	< 0.001
MDM2 (high vs low)	0.405	0.427–0.855	0.003	0.372	0.264–0.679	0.005

### Response to chemotherapy according to FKBP12 expression

Anthracyclines are among the most commonly used chemotherapeutic agents for breast cancer. However, only some patients respond well to anthracycline-based chemotherapy. Of the 524 invasive breast cancer patients, 166 received anthracycline-based neoadjuvant chemotherapy. We investigated further the correlation of FKBP12 expression with the efficacy of anthracycline-based neoadjuvant chemotherapy. We analyzed FKBP12 expression in biopsies taken at diagnosis and its relationship with pathologic complete remission (pCR), defined as the disappearance of invasive tumor lesion in surgically removed breast and axillary lymph nodes after chemotherapy. As shown in Table 3, tumor response to chemotherapy was significantly correlated with the expression status of FKBP12. Low FKBP12 expression patients had a significant lower rate of pCR. The pCR rate was significantly lower in the low FKBP12 expression group than that in the high FKBP12 expression group (*n *= 166, *P *= 0.006). Similarly, among the subtypes, FKBP12 loss correlated with lower pCR in TNBC patients (*n *= 67, *P *= 0.003), but not in patients with luminal (*n *= 65, *P *>0.05) or HER2+ breast cancer (*n *= 34, *P *>0.05). This observation was also especially prominent in the MDM2-positive subgroup (*P *<0.001 for MDM2-positive subgroup and *P *= 0.005 for MDM2-negative subgroup) (Table 3). These data further supported the above prognostic results (Figs. 2, 3), where anthracycline was also included in the adjuvant chemotherapy of most of the patients. In addition, in univariate and multivariate analyses, FKBP12 loss was an independent predictor against pCR for the overall population (*P *= 0.002 and *P *= 0.004, respectively) (Table 4) and TNBC patients (*P *= 0.003 and *P *= 0.009, respectively) (Table 5). Therefore, FKBP12 loss in breast cancer patients, especially in TNBC patients, was specifically associated with increased resistance to anthracycline-based chemotherapy.

**Table 3:** Correlation between chemoresponse and FKBP12 expression by intrinsic cancer subtype

Subtypes	*n*	PCR rate (FKBP12 high/low)	*P* value
All patients	166	47.0%/15.1%	0.006
Luminal (non HER2+)	65	33.8%/18.5%	0.412
HER2+	34	38.2%/17.6%	0.294
TNBC	67	64.2%/10.4%	0.003
TNBC = 67			
MDM2 high expression	45	71.1%/11.1%	< 0.001
MDM2 low expression	22	50.0%/9.10%	0.005

**Table 4:** Univariate and multivariate analyses of pCR against various characteristics in all patients

Variable	pCR rate (%)	Univariate analysis *P* value	Multivariate analysis *P* value
FKBP12 (high/low)	47.0/15.1	0.002	0.004
MDM2 (high/low)	21.4/33.8	0.038	0.065
Size (pT1-2/pT3-4)	27.2/29.4	0.484	0.507
Histological grade (I–II/III)	24.6/30.8	0.371	0.438
LN metastasis (posi/neg)	28.2/26.2	0.673	0.328
*NPI* (< 3.4/≥ 3.4)	21.4/32.4	0.039	0.107
MIB (< 10%/≥ 10%)	22.8/31.5	0.071	0.337
ERα (posi/neg)	22.5/36.1	0.023	0.045
PR (posi/neg)	19.7/35.4	0.028	0.047
HER2 amplification (posi/neg)	29.7/26.3	0.336	0.503
Molecular subtype (TNBC/others)	41.8/17.2	0.003	0.035

**Table 5:** Univariate and multivariate analyses of pCR against various characteristics in TNBC patients

Variable	pCR rate (%)	Univariate analysis *P* value	Multivariate analysis *P* value
FKBP12 (high/low)	64.2/10.4	0.003	0.009
MDM2 (high/low)	19.4/35.8	0.042	0.105
Size (pT1-2/pT3-4)	41.5/43.2	0.642	0.466
Histological grade (I–II/III)	39.5/44.7	0.407	0.637
LN metastasis (posi/neg)	45.3/39.1	0.373	0.578
*NPI* (< 3.4/≥ 3.4)	38.3/46.4	0.175	0.233
MIB (< 10%/≥ 10%)	36.4/52.8	0.043	0.153

### FKBP12 sensitizes breast cancer cells to chemotherapy

**Fig. 4:**
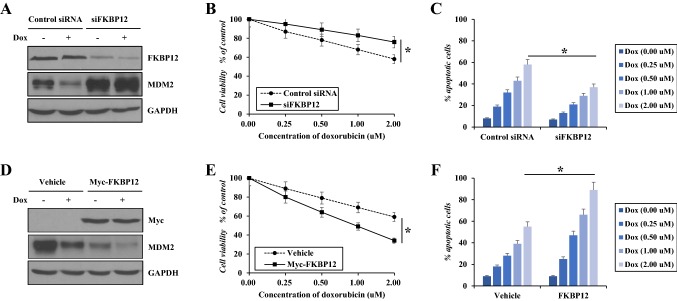
Effect of FKBP12 on cell response to doxorubicin. MDA-MB-468 cells were transfected with FKBP12 siRNA or Myc-tagged FKBP12, and then, the tumor cells were treated with different concentrations of doxorubicin for 24 h followed by western blot (**a**, **d**), cell viability (**b**, **e**), and apoptosis assay (**c**, **f**). Data represent mean ± SEM of three independent experiments. *P <0.05

We also investigated whether FKBP12 expression confers sensitivity to chemotherapy in breast cancer cells. We performed FKBP12 silence or transfection along with doxorubicin treatment in MDA-MB-468 cells and tested the MDM2 expression and cell survival. Western blot assay showed that the cellular expression of MDM2 was negatively regulated by FKBP12. FKBP12 silence led to significant upregulation of MDM2. Doxorubicin inhibited MDM2 expression in control MDA-MB-468 cells, but not in MDA-MB-468 cells with FKBP12 silence (Fig. 4a). Accordingly, MDA-MB-468 cells with FKBP12 silence were less responsive to doxorubicin-induced cytotoxic and apoptotic effect than control MDA-MB-468 cells (Fig. 4b, c). In contrast, in FKBP12-transfected MDA-MB-468 cells, MDM2 was more greatly inhibited by doxorubicin (Fig. 4d), resulting in greater cytotoxic and apoptotic effect (Fig. 4e, f).

## Discussion

MDM2 is a multifunctional oncoprotein and is overexpressed in various human cancers, including human breast cancer [13]. Overexpression of MDM2 in tumors correlates with disease progression and predicts a poor treatment outcome [14]. It has been well established that the oncogenic function of MDM2 protein is mainly through inhibiting the tumor suppressor p53 (restraining p53-mediated transcription and promoting p53 ubiquitination). The MDM2 gene is itself a transcript target of p53, and MDM2 and p53 form a mutual negative-feedback loop. When cells are in normal growing or in unstressed status, the MDM2-p53 negative-feedback loop is intact. However, in response to cellular stress and DNA damage such as treatment with certain chemotherapeutic drugs, p53 is rapidly accumulated and activated, inducing not only p21 and PUMA for cell-cycle arrest and apoptosis but also increased transcription of MDM2. This p53-induced increase in MDM2 expression inhibits p53, which is an important mechanism for the development of chemoresistance [15–18]. MDM2 also plays p53-independent roles. In the absence of functional p53, tumor cells that express high levels of MDM2 still show high invasive potential [19]. P53-independent activities and mechanisms of MDM2 have attracted increasing attention in recent years. A recent series of studies, including ours, indicate that MDM2 is able to interact with other cellular molecules involved in tumor promotion and metastasis, such as XIAP [20, 21], VEGF [22, 23], and Akt [24, 25].

Recently, we reported that FKBP12 interacted with MDM2 and induced MDM2 ubiquitination and degradation [12]. FKBP12-mediated degradation of MDM2 confers continuing and constitutive activation of p53, suppression of XIAP, and consequent sensitization of cancer cells to the cytotoxic and apoptotic effects of doxorubicin. P53 is a classic tumor suppressor, while XIAP is an important member of the inhibitor-of-apoptosis protein (IAP) family. Therefore, FKBP12-mediated degradation of MDM2 should be clinically significant. Cytotoxic chemotherapy, particularly anthracycline-based chemotherapy regimens, is currently the mainstay of treatment for human breast cancer. We herein examined the expression of FKBP12 by immunohistochemistry in breast cancer and analyzed its correlation with anthracycline efficacy. Results suggest that the expression level of FKBP12 in breast cancer tissue can serve as a predictive biomarker of therapeutic outcome. Low FKBP12 expression was specifically correlated with poor prognosis and increased resistance to anthracycline-based chemotherapy. Kaplan–Meier survival analysis showed that OS and DFS were both significantly lower in the low FKBP12 expression group than those in the high FKBP12 expression group. In patients treated with anthracycline-based preoperative chemotherapy, low FKBP12 expression patients had a significant lower rate of pCR. Importantly, these results seemed to be driven mainly by MDM2. These observations were especially prominent in the MDM2-positive subgroup. Finally, we performed FKBP12 silence or transfection in MDA-MB-468 breast cancer cells to confirm whether FKBP12 regulates MDM2 expression and confers sensitivity to chemotherapy. FKBP12 silence led to significant upregulation of MDM2. Accordingly, MDA-MB-468 cells with FKBP12 silence were less responsive to doxorubicin-induced cytotoxic and apoptotic effect than control MDA-MB-468 cells. In contrast, in FKBP12-transfected MDA-MB-468 cells, MDM2 was more greatly inhibited by doxorubicin, resulting in greater cytotoxic and apoptotic effect.

As mentioned, the MDM2-p53 feedback loop strongly operates leading to chemoresistance. In contrast, FKBP12-mediated degradation of MDM2 inhibits the MDM2-p53 feedback loop, resulting in sensitivity of cancer cells to anticancer treatment. Furthermore, suppression of XIAP following FKBP12-mediated MDM2 degradation represents an additional mechanism contributing to enhanced sensitivity. FKBP12-mediated MDM2 degradation and subsequent suppression of XIAP are p53-independent. Therefore, this mechanism may play roles in cancers with different p53 background. This is significantly important. It has been shown that most cancer patients, including breast cancer patients, do not carry wild-type p53 [26]. In addition, the incidence of p53 loss or mutation is obviously higher in aggressive cancer [27–29].

Although FKBP12 is widely expressed in normal cells/tissues, its expression in cancer cells/tissues has not been extensively studied. A recent study found that the expression level of FKBP12 varied significantly among different cancer cell lines [30]. We further observed a negative correlation between FKBP12 and MDM2 expression, which determined the responsiveness of cancer cells to anticancer treatment [12]. Here, we validated these observations in human breast cancer and indicated that FKBP12-MDM2 is predictive for therapeutic outcome. We provide a systematic investigation of the clinical relevance of FKBP12.

In summary, we propose that FKBP12 loss, which can be enhanced by MDM2 overexpression, predicts poor prognosis and chemoresistance. By future large prospective, randomized, controlled studies, the therapeutic strategy increasing the expression of FKBP12 may be a valuable addition to chemotherapy, especially in MDM2-overexpressed patients.

## Data Availability

The data sets used and/or analyzed during the current study are available from the corresponding author on reasonable request.

## Abbreviations

FKBP12: FK506-binding protein 12
MDM2: Mouse double minute 2
OS: Overall survival
DFS: Disease-free survival
TNBC: Triple-negative breast cancer
pCR: Pathologic complete response
mTOR: Mammalian target of rapamycin
RyR: Ryanodine receptor
TGF-βRI: Transforming growth factor-β type I receptor
EGFR: Epidermal growth factor receptor
AJCC: American Joint Committee on Cancer
NPI: Nottingham prognostic index
